# Spreading Advantages of Coresident Plasmids *bla*_CTX-M_-Bearing IncFII and *mcr-1*-Bearing IncI2 in Escherichia coli

**DOI:** 10.1128/spectrum.01706-21

**Published:** 2022-02-16

**Authors:** Kun He, Wenya Li, Bing Zhao, Hui Xu, Yushan Pan, Dandan He, Gongzheng Hu, Hua Wu, Li Yuan

**Affiliations:** a College of Veterinary Medicine, Henan Agricultural University, Zhengzhou, China; Dublin City University

**Keywords:** *bla*_CTX-M_, *mcr-1*, IncFII, IncI2, coresident

## Abstract

Two diverse conjugative plasmids can interact within bacterial cells. However, to the best of our knowledge, the interaction between *bla*_CTX-M_-bearing IncFII plasmid and *mcr-1*-carrying IncI2 plasmid colocated on the same bacterial host has not been reported. This study was initiated to explore the interaction and to analyze the reasons that these two plasmids are often coresident in multidrug-resistant Escherichia coli. To assess the interactions on plasmid stabilities, fitness costs, and transfer rates, we constructed two groups of isogenic derivatives, C600_FII_, C600_I2_, and C600_FII+I2_ of E. coli C600 and J53_FII_, J53_I2_, and J53_FII+I2_ of E. coli J53, respectively. We found that carriage of FII and I2 plasmids, independently and together, had not impaired the growth of the bacterial host. It was difficult for the single plasmid FII or I2 in E. coli C600 to reach stable persistence for a long time in an antibiotic-free environment, while the stability would be striking improved when they coresided. Meanwhile, plasmids FII and I2, whether together or apart, could notably enhance the fitness advantage of the host; moreover, E. coli coharboring plasmids FII and I2 presented more obvious fitness advantage than that carrying single plasmid FII. Coresident plasmids FII and I2 could accelerate horizontal cotransfer by conjugation. The transfer rates from a strain carrying coresident FII and I2 plasmids increased significantly when it mated with a recipient cell carrying one of them. Our findings highlight the advantages of coinhabitant FII and I2 plasmids in E. coli to drive the persistence and spread of plasmid-carried *bla*_CTX-M_ and *mcr-1* genes, although the molecular mechanisms of their coresidence warrant further study.

**IMPORTANCE** More and more *Enterobacteriaceae* carry both *bla*_CTX-M_ and *mcr-1*, which are usually located on IncFII-type and IncI2-type plasmids in the same bacterial host, respectively. However, the study on advantages of coresident plasmids in bacterial host is still sparse. Here, we investigated the stability, fitness cost, and cotransfer traits associated with coresident IncFII-type and IncI2-type plasmids in E. coli. Our results show that coinhabitant plasmids in E. coli are more stable, confer more fitness advantages, and are easier to transfer and cotransfer than a single plasmid IncFII or IncI2. Our findings confirm the advantages of coresident plasmids of *bla*_CTX-M_-bearing IncFII and *mcr-1*-bearing IncI2 in clinical E. coli, which will pose a serious threat to clinical therapy and public health.

## INTRODUCTION

So far, *bla*_CTX-M_ is still the most common extended-spectrum beta-lactamases (ESBLs) genotype in clinical *Enterobacteriaceae* worldwide; meanwhile, *mcr-1* gene is the first confirmed movable colistin resistance (*mcr*) gene (1, 2). In addition, the worrisome situation is that conjugative plasmids act as the major vehicle involved in the transmission of *bla*_CTX-M_ and *mcr-1* genes (1, 3). Among them, IncF-type conjugative plasmids, especially the IncFII-replicon, play an important role in the global dissemination of *bla*_CTX-M_-type ESBLs (4–6). Similarly, the IncI2-type conjugative plasmid is the most epidemiological successful vector for horizontal spread of *mcr-1* (1, 7–9).

Recently, resistant genes *bla*_CTX-M_ and *mcr-1* have been simultaneously detected in *Enterobacteriaceae* species isolated from humans and animals (8, 10, 11). Although they sometimes coexist on the same plasmid (8, 12), the *bla*_CTX-M_ and *mcr-1* genes are usually located on diverse plasmids of the same bacterial host (13–15). In a previous survey on antimicrobial-resistant bacterial isolates in China, Escherichia coli LWY24 was isolated from healthy chicken feces in Henan Province (15). The isolate LWY24 harbored *bla*_CTX-M-55_ and *mcr-1*, which were located on an IncFII replicon pLWY24J-3 (*bla*_CTX-M-55_-bearing, MN702385) and an IncI2 replicon pLWY24Jmcr-1 (*mcr-1*-carrying, MN689940), respectively, and could carry out horizontal transfer by plasmid conjugation, independently or simultaneously.

Previous studies confirmed that two distinct conjugative plasmids could interact within bacterial cells (16–20). To the best of our knowledge, the interaction between *bla*_CTX-M_-bearing IncFII plasmid and *mcr-1*-carrying IncI2 plasmid colocated on the same cell has not been reported. This study was initiated to explore the characteristics of coresident IncFII-type and IncI2-type plasmids in E. coli.

## RESULTS AND DISCUSSION

### The plasmids FII and I2 had no effect on bacteria growth.

**FIG 1:**
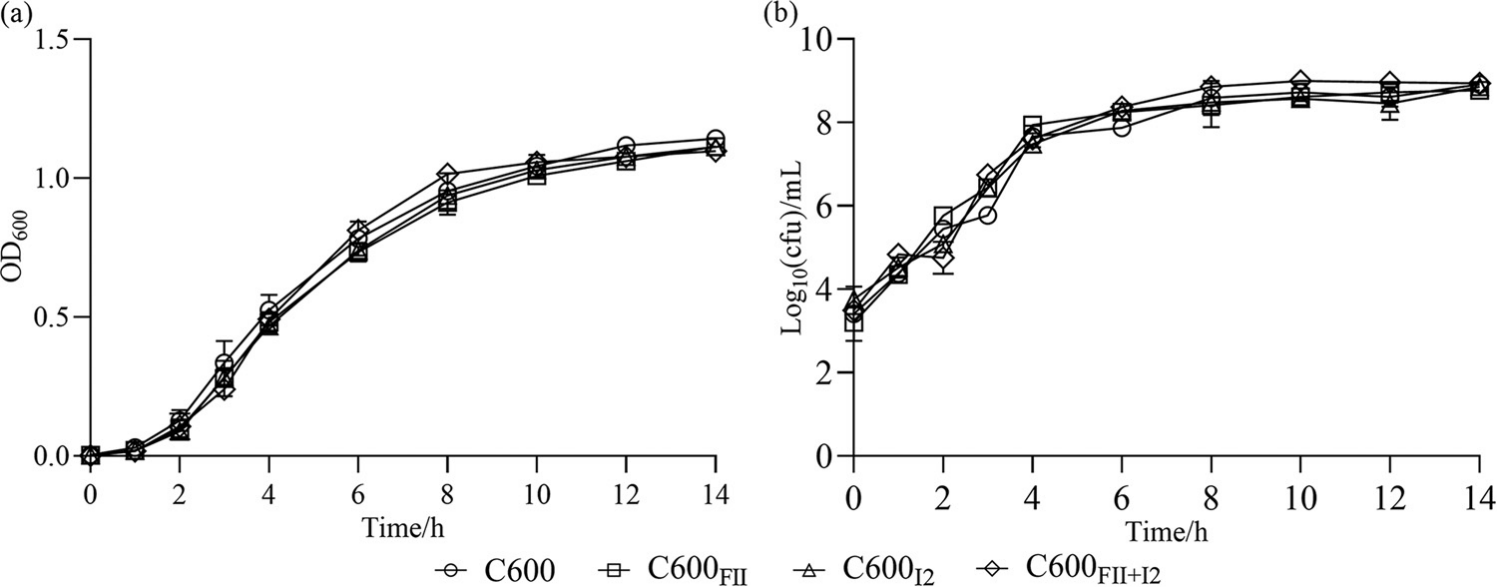
Growth curves of E. coli C600 and its isogenic derivatives. (a) OD_600_ values for each time point. (b) CFU values. The strains C600_FII_, C600_I2_, and C600_FII+I2_ were isogenic derivatives of E. coli C600, which harbored the plasmids, FII and/or I2 (abbreviations of plasmids pLWY24J-3 and pLWY24Jmcr-1, respectively). Curve indicates the mean of results from three independent experiments and error bars denote standard deviations for each time point.

Growth kinetics of the four isogenic strains were plotted respectively based on optical density at 600 nm (OD_600_) values and the log_10_ CFU values after 14-h assessment (Fig. 1). No obvious difference in growth was observed among bacteria, which indicated that carriage of FII and I2 plasmids, independently and together, had not impaired the growth of the bacterial host. The results coincided with previous studies on *bla*_CTX-M_-carrying IncFII plasmid (9) and *mcr-1*-harboring IncI2 plasmid (7), which further explained why IncFII-replicon and IncI2-replicon plasmids were dominant in *bla*_CTX-M_-carrying and *mcr-1*-harboring E. coli strains, respectively (6, 9).

### Coresident plasmids FII and I2 improved stabilities.

**FIG 2:**
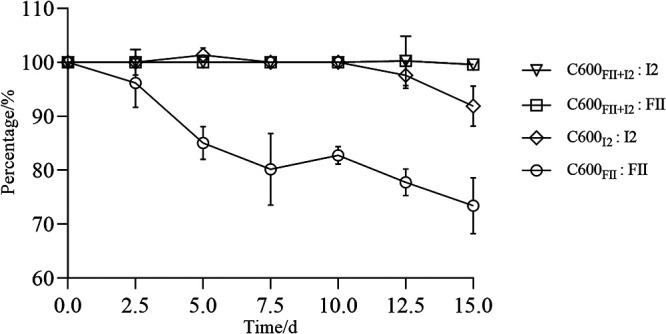
*In vitro* stability of plasmids pLWY24J-3 and pLWY24Jmcr-1. The plasmids pLWY24J-3 and pLWY24Jmcr-1 are abbreviated FII and I2, respectively. The strains C600_FII_, C600_I2_, and C600_FII+I2_ were isogenic derivatives of E. coli C600, which harbored the plasmids, FII and/or I2. Data shown are the means of results from three independent assays and error bars represent the standard deviation of the mean (*n* = 3).

In order to analyze the stability of acquired plasmids over time in the absence of selection, we propagated the isogenic strains, C600_FII_, C600_I2_, and C600_FII+I2_, in antibiotic-free culture medium for 15 days (i.e., ∼150 generations) (Fig. 2). The results demonstrated that the single plasmid FII in the host could not be stably maintained. It was partially lost from day 2.5, and only about 73.5% remained at day 15. Meanwhile, the plasmid I2 loss occurred from day 12.5, with a total loss of about 8.1% at the end. From this, the stability of two plasmids was decreased in different degrees in the absence of antibiotic selective pressure, especially that of plasmid FII.

Intriguingly, when plasmids FII and I2 coresided in the host, almost no plasmid loss was detected over the 15 days of the experiment, which implied that the stability of *bla*_CTX-M_-carrying FII plasmid and *mcr-1*-harboring I2 plasmid could significantly improve by coresidence. The results were consistent with previous studies that the stability of coexistent plasmids increased (17), which contributed to elucidating why *bla*_CTX-M_ and *mcr-1* are easier to locate on diverse plasmids in the same bacterial host worldwide (13–15).

### Cells coharboring plasmids FII and I2 presented fitness advantages.

**FIG 3:**
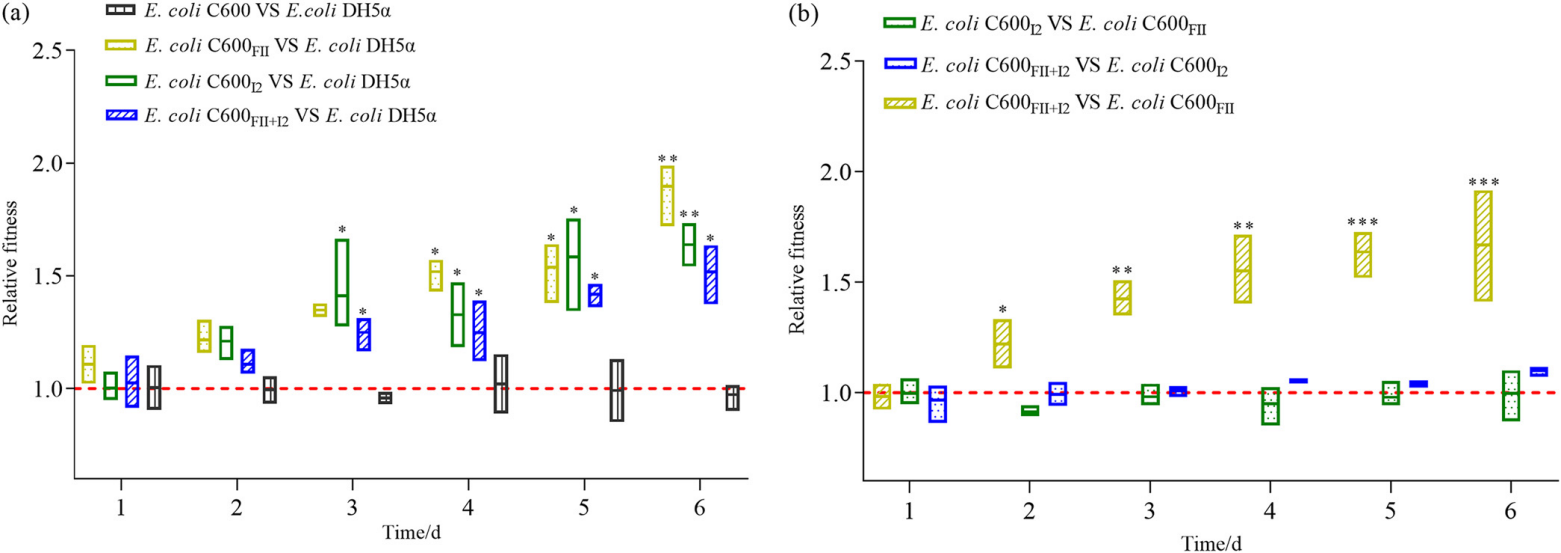
Pairwise competition *in vitro* between E. coli C600, E. coli DH5α, C600_FII_, C600_I2_, and C600_FII+I2_ normalized to a 50/50 starting ratio. (a) Relative fitness of E. coli C600, C600_FII_, C600_I2_, and C600_FII+I2_ against E. coli DH5α, respectively. (b) Pairwise competition of C600_FII_, C600_I2_, and C600_FII+I2_. The strains C600_FII_, C600_I2_, and C600_FII+I2_ were isogenic derivatives of E. coli C600, which harbored the plasmids, FII and/or I2 (abbreviations of plasmids pLWY24J-3 and pLWY24Jmcr-1, respectively). The pairwise strains were competed in LB broth medium for 6 days, with six passages. Each boxplot represents the distribution of relative fitness values for each time point: the horizontal line in the box is the median, and the bottom and top of the box are the lowest and the highest values. Asterisks denote significant differences using unpaired Student’s *t* test (*, *P < *0.05; **, *P < *0.01; ***, *P < *0.001).

To determine the relative carriage costs of plasmids FII and I2, pairwise competitions were carried out between the plasmid-free strain E. coli DH5α and three isogenic derivatives, C600_FII_, C600_I2_, and C600_FII+I2_. The outcome competition revealed that there were no fitness costs between plasmid-free strains E. coli DH5α and E. coli C600 (Fig. 3a). However, the plasmid-harboring strains presented high fitness advantages in comparison with E. coli DH5α, which obviously increased over time (Fig. 3a). The isogenic strains, C600_I2_, C600_FII+I2_, and C600_FII_, significantly outcompeted E. coli DH5α from day 3 (relative fitness [RF] = 1.28 ± 0.0085, *P = *0.0443), 3 (RF = 1.29 ± 0.021, *P = *0.0166), and 4 (RF = 1.56 ± 0.05, *P = *0.0465), respectively. Thus, plasmids FII and I2, whether together or apart, bestowed the fitness advantages on the host bacteria, which were consistent with some previous reports (7, 21) and contributed to the plasmids gradually becoming the capital vehicles for *bla*_CTX-M_ and *mcr-1* horizontal disseminations (6, 22).

Thereafter, the results of plasmid-plasmid competition assays described that no fitness costs were observed between C600_I2_ and C600_FII+I2_ after 144-h assessments (Fig. 3b), indicating that plasmid FII would not incur any additional fitness costs on its host cell when transferred to strain C600_I2_. In contrast, strain C600_FII+I2_ showed significant competition advantages over strain C600_FII_ from 48 h (RF = 1.22 ± 0.09, *P = *0.0317) to 144 h (RF = 1.67 ± 0.09, *P = *0.0008), demonstrating that plasmid I2 could further decrease fitness costs on its host when entering strain C600_FII_ (Fig. 3b). Together, E. coli coharboring plasmids FII and I2 will not confer more fitness costs than bacteria carrying one plasmid FII or I2, which helps to promote the coexistence of plasmid-carried *bla*_CTX-M_ and *mcr-1* in E. coli and to clarify why more and more *Enterobacteriaceae* carry both *bla*_CTX-M_ and *mcr-1* (11, 15).

### Coinhabitant plasmids FII and I2 contributed to cotransfer.

The transfer speeds of plasmids FII and I2 were analyzed by serial transfer experiments (Table 1). The plasmid FII exhibited higher transfer speeds than plasmid I2. Furthermore, whether the plasmids FII and I2 coexisted in the same cell or in different cells, the transconjugants, coharboring plasmids FII and I2, could be obtained, but this was easier and faster in the former situation, which partially explained why isolates more often harbored multiple plasmids. These results proved that coresident plasmids FII and I2 in E. coli could accelerate horizontal cotransfer.

**TABLE 1:** The transfer speeds of plasmids pLWY24J-3 and pLWY24Jmcr-1, independently and together, in diverse settings^*a*^

Donor	Recipient	Transconjugant	Conjugation time (min)				
			0	5	10	20	60
C600_FII_	E. coli J53	TJ_FII_	+++	+++	+++	+++	+++
C600_I2_	E. coli J53	TJ_I2_	+	++	++	++	+++
C600_FII+I2_	E. coli J53	TJ_FII_	+++	+++	+++	+++	+++
	E. coli J53	TJ_I2_	0	++	++	++	+++
	E. coli J53	TJ_FII+I2_	0	0	+	+	+
C600_FII_/C600_I2_ (1:1)	E. coli J53	TJ_FII_	+++	+++	+++	+++	+++
	E. coli J53	TJ_I2_	0	+	+	+	++
	E. coli J53	TJ_FII+I2_	0	0	0	0	+

a The plasmids pLWY24J-3 and pLWY24Jmcr-1 are abbreviated FII and I2, respectively. The strains C600_FII_, C600_I2_, and C600_FII+I2_ were isogenic derivatives of E. coli C600, which harbored the plasmids FII and/or I2. “+” represents that there were 1 to 10 transconjugants on all LB agar plates supplemented with colistin and/or cefotaxime, “++” represents 11 to 99 transconjugants, “+++” represents ≥100 transconjugants, and “0” represents no transconjugants. The naming of transconjugants is as follows: capital “T” stands for the transconjugant, capital “J” represents the recipient, and subscript of transconjugants represents the plasmid which was transferred from the donor to the transconjugant.

**FIG 4:**
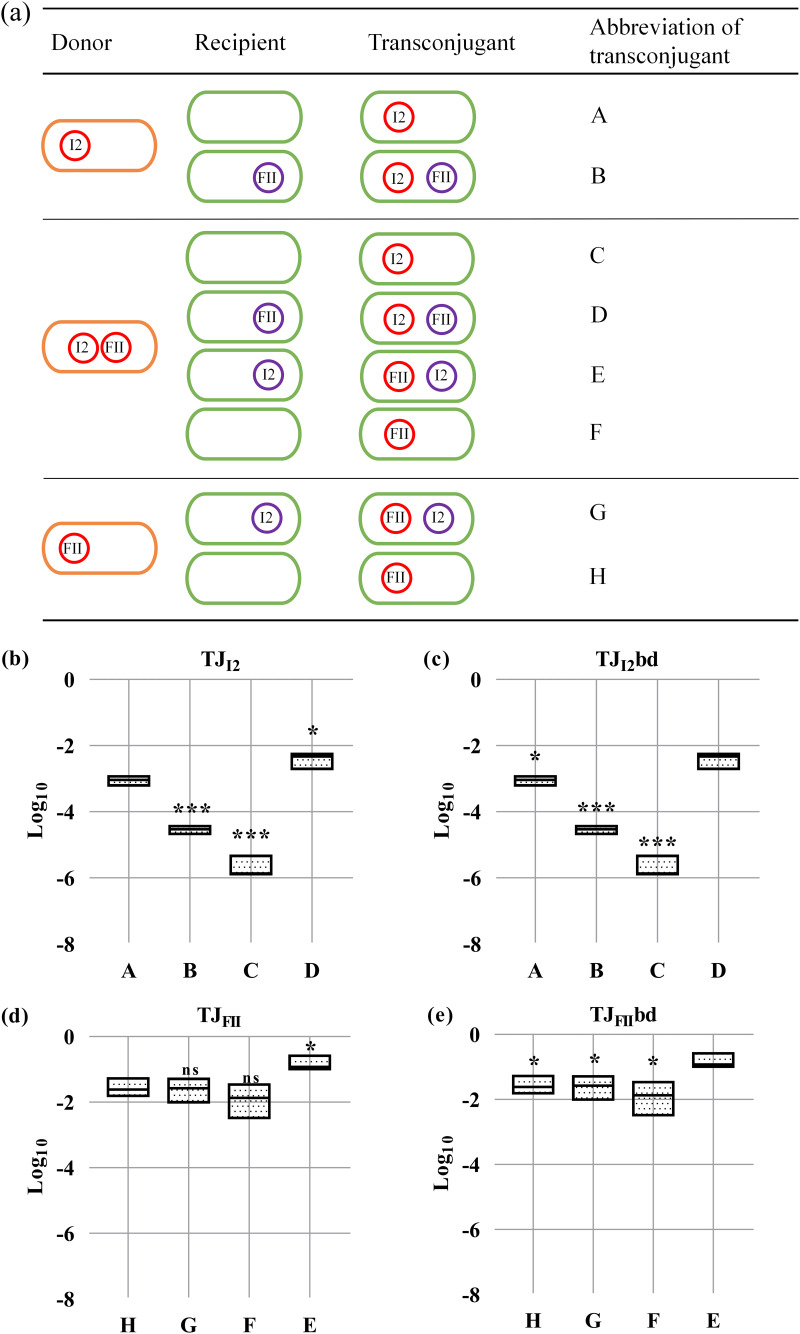
The transfer interactions between plasmids pLWY24J-3 and pLWY24Jmcr-1. The plasmids pLWY24J-3 and pLWY24Jmcr-1 are abbreviated FII and I2, respectively. (a) A diagram of distinct donors, recipients, and transconjugants in the dual conjugation assays. Orange ellipse represents E. coli C600, green ellipse represents E. coli J53, and small circles in ellipses represent plasmids FII and/or I2. (b and c) The transfer rates of plasmid I2. (d and e) The transfer rates of plasmid FII. Control groups are indicated in the title of each plot. Each boxplot represents the distribution of transfer rate values: the horizontal line in the box is the median, and the bottom and top of the box are the lowest and the highest values. Asterisks denote significant differences using unpaired Student’s *t* test (*, *P < *0.05; **, *P < *0.01; ***, *P < *0.001; ns, not significant).

We measured the conjugation rates of eight pairs of plasmids (Fig. 4). First, we compared the mating rates of each plasmid when in the presence of a coresident plasmid with its own mating rates to the mating rates of each plasmid when alone in the donor cell (Fig. 4b and d). The conjugation rates of I2 plasmid in the donor C600_FII+I2_ were severely decreased, approximately 141-fold lower than those in the donor C600_I2_, while there was no significant difference in the conjugation rates of FII plasmid between the donors C600_FII+I2_ and C600_FII_. We also examined the conjugation frequencies of single plasmids in matings to recipient cells carrying another plasmid (Fig. 4b and d). Similar to the above results, the conjugation rates of plasmid I2 were also significantly decreased, by 31.1-fold, when recipient cells harbored plasmid FII compared with those when the recipient was plasmid-free. These results implied that plasmid FII could inhibit the transfer of plasmid I2 whether plasmid FII presented in the donor or in the recipient, while plasmid I2 had no effect on the transfer of plasmid FII in the similar case.

Further, we explored the conjugation frequencies of each plasmid in the presence of a coresident plasmid in donor cells mating with recipient cells carrying another plasmid (Fig. 4c and e). Surprisingly, the conjugation rates of I2 and FII plasmids in this situation all increased significantly compared with those of the other situations. We speculate that transfer of the other plasmid will possibly increase significantly when the donor contains two plasmids and the recipient contains one of the plasmids. A previous study reported that negative interactions were significantly more frequent when plasmids occupied the same cell (18). Meanwhile, our above results also demonstrated that plasmid FII could inhibit the transfer of plasmid I2 when the plasmids were coresident in the donor, although further studies are needed to verify the reason this happens.

Many factors could affect plasmid stability, fitness cost, and conjugation, such as sizes, copy numbers, and replications (17, 23, 24). In this study, the plasmids FII and I2 were of comparable size (Table 2) and conferred similar effects on the host cell. It is worth noting that the plasmids FII and I2 present positive interactions, which appears to be different from the positive epistasis between small and large plasmids described by Millan et al. (17), indicating that positive interactions may be related to the plasmid nature but not to the plasmid size. Meanwhile, we conducted the plasmid copy numbers by quantitative real-time PCR according to the previous study with some modifications (17, 25, 26). The results demonstrated that the copy numbers of plasmid FII and I2 per chromosome were 1.01 ± 0.11 and 2.0 ± 0.15, respectively, while those of coinhabitant plasmids FII and I2 significantly declined to 0.84 ± 0.31 copies per cell (FII, *P = *0.002) and 1.69 ± 0.20 (I2, *P = *0.001) copies per cell. Although IncFII and IncI2 plasmids are low-copy-number conjugative plasmids (4), their copy numbers further decrease when they coexist in the same host, which partially explains improved persistence, better adaptability, and easier transfer of coinhabitant plasmids FII and I2. Further studies are needed to analyze other factors.

**TABLE 2:** Conjugative plasmids used in this study

Plasmids	Group	Size (kb)	Abbreviation	Resistance genes	Origin	Yr	Reference	Accession no.
pLWY24J-3	IncFII, F33:A−:B−	68.72	FII	*bla*_CTX-M-55,_ *bla*_TEM-1b,_ *rmtB*	E. coli LWY24	2016	15	MN702385
pLWY24Jmcr-1	IncI2	62.01	I2	*mcr-1*	E. coli LWY24	2016	15	MN689940

**Conclusion.** In conclusion, compared with single plasmids carried in E. coli, coinhabitant IncFII-type and IncI2-type plasmids in E. coli were stably persistent, conferred more fitness advantages, and were easier to transfer and cotransfer. Our findings highlight the advantages of coresident IncFII-type and IncI2-type plasmids in E. coli to drive the persistence and spread of plasmid-carried *bla*_CTX-M_ and *mcr-1*, which could pose a serious threat to clinical therapy and public health.

## MATERIALS AND METHODS

### Bacterial strains and plasmids.

We used the following bacterial strains: azide-resistant E. coli J53, rifampin-resistant E. coli C600, and E. coli DH5α. The conjugative plasmids, pLWY24J-3 (*bla*_CTX-M-55_-bearing, 68.72 kb, IncFII, F33:A−:B−, abbreviated FII) and pLWY24Jmcr-1 (*mcr-1*-carrying, 62.01 kb, IncI2 replicon, abbreviated I2), were obtained from one multidrug-resistant isolate, E. coli LWY24 O3:H25, ST93, from chicken in China (15) and were used to transform E. coli C600 or E. coli J53 by electrotransformation, independently and together, and to generate two groups of isogenic derivatives, designated C600_FII_, C600_I2_, and C600_FII+I2_ and J53_FII_, J53_I2_, and J53_FII+I2_, respectively. The strains and conjugative plasmids used in the study are detailed in Table 2 and 3.

**TABLE 3:** Strains used in this study

Strains	Description and characteristics	Reference	Accession no.
E. coli LWY24	O3:H25-ST93, isolated from chicken, conferred resistance to cefotaxime, gentamicin, amikacin, oxytetracycline, doxycycline, florfenicol, colistin, enrofloxacin, fosfomycin, and sulfamonomethoxine/trimethoprim.	15	CP054556
E. coli DH5α	Used as a reference strain for fitness assays *in vitro*.	7	
E. coli C600	Rifampin resistance, plasmid-free, used as a recipient to construct isogenic derivatives, C600_FII_, C600_I2_, and C600_FII+I2_, and used as a reference strain for growth kinetics assays.	This study	
E. coli J53	Azide resistance, plasmid-free, used as a recipient to construct isogenic derivatives, J53_FII_, J53_I2_, and J53_FII+I2_, and used as a recipient for transfer experiments.	This study	
C600_FII_, C600_I2_, and C600F_II+I2_	Isogenic derivatives of E. coli C600, which harbored the plasmids FII and/or I2.	This study	
J53F_II_, J53_I2_, and J53F_II+I2_	Isogenic derivatives of E. coli J53, which harbored the plasmids FII and/or I2.	This study	

### Growth kinetics and plasmid stability.

Growth curves for E. coli C600 and its isogenic strains, C600_FII_, C600_I2_, and C600_FII+I2_, were established. After overnight incubation at 37°C, the cultures were inoculated (1:1,000 dilution) into four tubes containing 5 mL of fresh Luria-Bertani (LB) medium with shaking and grown to an OD_600_ of 0.5. Then, the cultures were diluted (1:1,000) in 60 mL of preheated fresh LB broth and inoculated at 37°C and 180 rpm. The OD_600_ values were measured at intervals (0 h, 1 h, 2 h, 3 h, 4 h, 6 h, 8 h, 10 h, 12 h, and 14 h) using a UV spectrophotometer. Meanwhile, the culture broths were serially diluted with 0.9% saline and plated onto LB agar plates. CFU were counted after 18 h of incubation at 37°C, and the log_10_ CFU values were calculated. Three independent biological replicates were performed.

To investigate plasmid stability, we propagated the isogenic strains, C600_FII_, C600_I2_, and C600_FII+I2_, in antibiotic-free LB broth for 15 days, diluting the cultures (1:1,000) every 12 h, as described previously (7, 27, 28). Periodically, the culture broths were serially diluted in 0.9% saline and plated onto LB agar. Colonies from each viable count were replica plated onto LB agar plates containing 4 mg/L of cefotaxime and/or 2 mg/L colistin and were randomly selected for confirmation of the presence of *bla*_CTX-M_ and/or *mcr-1* and the corresponding replicon typed by PCR.

### *In vitro* fitness assays.

To determine the fitness costs associated with bearing the plasmids FII and I2, both together and apart, four strains, E. coli DH5α, C600_FII_, C600_I2_, and C600_FII+I2_, were competed in fresh LB broths according to the method described in previous studies with some modifications (16, 25). The experiment was set up with a full-factorial design so that each strain was competed against every other strain. Meanwhile, we also compared the fitness advantages of plasmid-free E. coli DH5α and E. coli C600. To initiate growth competition, each overnight culture was inoculated in fresh LB medium and grown to an OD_600_ of 0.5, mixed in pairs at a ratio of 1:1, 10^−3^ diluted into LB broth, and grown for 24 h. Then, the mixture was again diluted 10^−3^-fold into fresh LB broth. This procedure was repeated until the competition experiment had lasted for 144 h (6 cycles). The total number of bacteria was determined by spreading properly diluted samples of each competition mixture on LB agar containing 4 mg/L of cefotaxime and/or 2 mg/L colistin at 0 h, 24 h, 48 h, 72 h, 96 h, 120 h, and 144 h. Ten colonies per plate were randomly selected for confirmation according to the above description.

The relative fitness (RF) was calculated as follows according to the methods described previously (7, 17), using the formula RF = (log_10_S1_d_*_t_* − log_10_S1_d0_)/(log_10_S2_d_*_t_* − log_10_S2_d0_), where S1_d_*_t_*, S1_d0_, S2_d_*_t_*, and S2_d0_ are the respective CFU densities of the strains and *t* is time in days. If RF is not equal to 1, there exists a fitness difference between the competitors, that is, RF > 1 indicates that there exists a fitness advantage, whereas RF < 1 represents a fitness cost. Statistical analysis was carried out via the software GraphPad Prism 8.0 (GraphPad Software Inc., La Jolla, CA).

### Serial transfer experiments.

To compare the relative transfer speeds of the plasmids FII and I2, the retransfer experiments were carried out using isogenic derivatives of E. coli C600 as the donor and E. coli J53 as the recipient. The donors were three settings as follows: (i) the donor cell was C600_FII_ or C600_I2_, (ii) the donor cell was C600_FII+I2_, or (iii) the donor cell was composed of a 1:1 mixture of C600_FII_ and C600_I2_. Standing overnight cultures were diluted 1:1,000 in fresh LB broth and incubated with shaking at 37°C to an OD_600_ of 0.5 and then mixed in pairs in a 4.0 mL total volume at a ratio of 1:1 and mated for 0 min, 5 min, 10 min, 20 min, and 60 min at 37°C. Matings were stopped by cooling the samples on ice for 1 min. Next, the samples were centrifuged at 4°C and 1,000 rpm for 10 min, 3.5 mL of supernatants was discarded, and then the residues were vigorously vortexed for 1 min to remix evenly. The mixtures (100 μL per plate) were plated on 5 agar plates supplemented with colistin and/or cefotaxime. The plates were subsequently incubated overnight at 37°C to allow colony formation and to count. Ten colonies per plate were randomly chosen for confirmation as described above. Experiments were repeated in three separate assays.

To compare intracellular and intercellular interactions between the plasmids FII and I2 on transfer efficiencies, the dual conjugation assays were further performed using isogenic derivatives of E. coli C600 as the donor and E. coli J53 or its isogenic derivatives as the recipient (Fig. 4a). The five settings were as follows: (i) the donor cell was C600_FII_ or C600_I2_ and the recipient cell was E. coli J53, (ii) the donor cell was C600_FII_ and the recipient cell was J53_I2_, (iii) the donor cell was C600_I2_ and the recipient cell was J53_FII_, (iv) the donor cell was C600_FII+I2_ and the recipient cell was E. coli J53, or (v) the donor cell was C600_FII+I2_ and the recipient cell was J53_FII_ or J53_I2_. Similar to the above procedures of transfer experiments, the donor and the recipient mixed at a ratio of 1:1 and mated for 4 h at 37°C. Then, the mixtures were serially diluted in 0.9% saline and plated on selective LB agar, and 10 colonies per plate were randomly chosen for confirmation as described above. At least three independent biological replicates were included for each sample. Finally, mating efficiencies were calculated and evaluated by statistical analyses.
